# Supplementary material for: A Likelihood Approach for Real-Time Calibration of Stochastic Compartmental Epidemic Models
Source: PLoS Comput Biol. 2017 Jan 17;13(1):e1005257. doi: 10.1371/journal.pcbi.1005257 (PMC5240920; doi:10.1371/journal.pcbi.1005257)
Supplement: S1 File — (TAR.GZ) [file pcbi.1005257.s014.tar.gz › HSPH_Online-SI-Revision/MSS10/n1-mild/n1-mild_NEW_table-8.pdf]

1	R0	{1.27677, 7.89375, 1.}	{1.85991, 13.7033, 0.56}	{{1.39882, 8.35044, 0.}, {2.2277, 18.8228, 2.}}	{46.1027, 76.9813, 70.}	inf	9.	16.8845	{4.86164, 32.3287}	105.609	0.307224	newly	11.	10.43	{3., 19.}	42.4545	0.225725	Reff	1.2423	1.79315	{1.37147, 2.11318}	44.7641	ar	400.	699.78	{533., 850.}	84.8
2	R0	{1.33231, 10.5259, 2.}	{1.33373, 5.5985, 0.72}	{{1.22544, 3.07157, 0.}, {1.41695, 6.88118, 2.}}	{7.00669, 50.8306, 72.}	inf	43.	30.1476	{8.64175, 47.5901}	46.0248	0.306881	newly	32.	37.96	{14., 60.}	47.6875	0.204446	Reff	1.07517	1.091	{1.02447, 1.14506}	5.47927	ar	467.	409.21	{199., 584.}	27.2827
3	R0	{1.2273, 6.76944, 1.}	{2.07761, 3.61891, 0.08}	{{1.24751, 2.12856, 0.}, {2.96265, 5.42517, 0.}}	{71.1208, 50.4543, 100.}	inf	19.	15.6372	{3.91327, 30.2849}	44.6302	0.276977	newly	19.	101.67	{19., 207.}	442.263	0.609995	Reff	1.19048	2.01969	{1.21164, 2.87931}	71.325	ar	314.	698.88	{192., 935.}	138.777
4	R0	{1.41215, 17.5742, 3.}	{1.26117, 6.17267, 0.93}	{{1.06738, 2.19254, 0.}, {1.6004, 16.157, 3.}}	{17.3716, 65.8321, 73.6667}	inf	19.	14.1605	{3.48104, 26.8817}	45.636	0.306775	newly	6.	28.02	{5., 65.}	375.333	0.53674	Reff	1.34578	1.20696	{1.0218, 1.5246}	16.8764	ar	465.	312.18	{38., 643.}	50.4731
5	R0	{1.42222, 6.15886, 0.1}	{1.45562, 5.15768, 0.39}	{{1.21159, 3.02112, 0.}, {2.01902, 13.1443, 2.}}	{17.7856, 57.6264, 444.}	inf	13.	19.85	{3.62057, 48.9806}	92.8386	0.31107	newly	24.	33.85	{7., 63.}	70.625	0.272031	Reff	1.34115	1.35657	{1.15176, 1.83349}	16.5664	ar	490.	408.63	{78., 829.}	43.6796
6	R0	{1.24363, 9.95596, 0.1}	{1.99205, 11.7044, 0.7}	{{1.18158, 6.00596, 0.}, {2.80387, 18.0404, 2.}}	{62.2654, 36.2734, 700.}	inf	3.	4.22021	{1.51151, 8.56704}	70.7498	0.223949	newly	3.	4.92	{0., 12.}	115.333	0.404369	Reff	1.23741	1.97862	{1.17641, 2.77004}	61.9669	ar	411.	588.43	{6., 927.}	92.309
7	R0	{1.36478, 5.96056, 1.}	{1.57464, 5.32792, 0.45}	{{1.06738, 2.19254, 0.}, {2.58092, 14.8279, 2.}}	{27.5308, 62.7365, 103.}	inf	14.	16.3106	{4.24984, 30.825}	59.6645	0.268307	newly	23.	44.61	{7., 82.}	129.	0.337595	Reff	1.32384	1.52103	{1.04274, 2.48057}	26.7887	ar	484.	460.24	{39., 902.}	53.6116
8	R0	{1.36335, 17.6429, 2.}	{1.1731, 17.7723, 0.22}	{{1.0529, 15.6337, 0.}, {1.35081, 19.5412, 1.}}	{13.9803, 7.75797, 89.}	inf	13.	3.6946	{1.39156, 7.63725}	71.58	0.637931	newly	3.	1.44	{0., 4.}	64.	0.649657	Reff	1.33472	1.15923	{1.04192, 1.33005}	13.2897	ar	484.	149.45	{10., 460.}	70.4277
9	R0	{1.26575, 5.28707, 0.1}	{1.24435, 4.06812, 0.39}	{{1.06738, 2.19254, 0.}, {1.28596, 3.98513, 1.}}	{5.90553, 46.75, 444.}	inf	7.	12.5811	{2.3184, 27.0849}	112.301	0.321861	newly	8.	24.62	{5., 47.}	223.	0.443728	Reff	1.13918	1.11239	{0.96399, 1.16398}	5.85916	ar	472.	291.58	{107., 459.}	42.822
10	R0	{1.42942, 9.49905, 2.}	{1.61019, 12.4225, 1.5}	{{1.28596, 3.98513, 0.}, {1.99753, 19.2013, 3.}}	{17.237, 49.8938, 46.}	inf	25.	39.9954	{12.3042, 63.4233}	77.3778	0.257288	newly	22.	25.87	{16., 36.}	31.9545	0.126881	Reff	1.26932	1.40194	{1.15915, 1.71372}	14.7295	ar	468.	599.	{333., 792.}	39.3761
11	R0	{1.46827, 15.0983, 0.1}	{1.19048, 13.0625, 1.86}	{{1.01011, 8.14942, 0.}, {1.44158, 15.4344, 3.}}	{22.0725, 18.79, 1806.}	inf	7.	4.68098	{1.60151, 7.94425}	53.8824	0.332968	newly	4.	2.84	{0., 6.}	58.5	0.477694	Reff	1.43009	1.16179	{0.988917, 1.41135}	21.7678	ar	495.	170.12	{22., 581.}	74.4808
12	R0	{1.25751, 13.279, 1.}	{1.30837, 13.5178, 2.59}	{{1.10202, 5.30671, 0.}, {1.50403, 17.9732, 4.}}	{13.42, 27.5001, 183.}	inf	9.	8.06969	{2.15437, 17.8038}	63.0659	0.336458	newly	6.	5.48	{1., 11.}	78.	0.479853	Reff	1.20847	1.25767	{1.06579, 1.4546}	13.0083	ar	366.	317.2	{36., 626.}	60.224
13	R0	{1.3425, 9.16361, 1.}	{1.24142, 4.28937, 0.59}	{{1.06738, 2.19254, 0.}, {1.28596, 5.50828, 2.}}	{11.2085, 65.2681, 109.}	inf	36.	19.7281	{3.89421, 33.0154}	56.8435	0.436633	newly	33.	33.81	{9., 61.}	52.9394	0.275503	Reff	1.12233	1.05615	{0.916965, 1.11454}	9.01889	ar	442.	323.51	{147., 497.}	35.9072
14	R0	{1.18936, 6.20583, 0.1}	{2.27946, 14.3246, 1.09}	{{1.78373, 9.06807, 0.}, {2.86501, 19.0355, 2.}}	{91.6546, 133.861, 1034.}	inf	20.	40.7593	{20.7988, 63.0674}	109.45	0.30032	newly	20.	26.66	{14., 41.}	47.4	0.171484	Reff	1.1287	2.11052	{1.68629, 2.5955}	86.9862	ar	382.	830.04	{707., 921.}	117.419
15	R0	{1.27137, 9.78868, 0.1}	{1.42021, 5.1012, 0.35}	{{1.06738, 2.5149, 0.}, {2.04423, 14.1631, 2.}}	{18.0641, 67.7982, 410.}	inf	14.	19.5668	{2.77287, 42.2314}	84.6676	0.315696	newly	12.	34.38	{10., 63.}	197.167	0.434242	Reff	1.20017	1.32613	{1.02071, 1.87222}	16.7269	ar	496.	387.29	{64., 794.}	45.1552
16	R0	{1.23102, 13.6452, 1.}	{1.96659, 14.293, 0.47}	{{1.63746, 10.1332, 0.}, {2.24242, 18.2464, 1.}}	{59.7526, 21.1643, 67.}	inf	23.	31.643	{15.8304, 46.792}	51.278	0.178203	newly	11.	19.43	{10., 30.}	81.7273	0.240479	Reff	1.16085	1.83502	{1.53772, 2.06983}	58.0755	ar	445.	769.21	{651., 867.}	72.8562
17	R0	{1.27213, 13.7452, 3.}	{2.13406, 14.2976, 0.34}	{{1.51691, 9.63533, 0.}, {2.88523, 18.8857, 1.}}	{68.1815, 23.3081, 88.6667}	inf	8.	11.213	{3.07115, 20.1693}	70.152	0.24888	newly	5.	7.9	{2., 13.}	96.4	0.324928	Reff	1.2505	2.08917	{1.49283, 2.82962}	67.474	ar	367.	762.98	{532., 927.}	117.995
18	R0	{1.25318, 11.2151, 0.1}	{1.31637, 9.34703, 1.72}	{{1.08063, 3.02112, 0.}, {1.39972, 14.758, 3.}}	{8.71838, 50.0721, 1696.}	inf	12.	11.3266	{3.63197, 17.551}	35.2843	0.191426	newly	5.	12.42	{4., 26.}	156.8	0.348801	Reff	1.17924	1.23932	{1.02609, 1.31906}	8.65217	ar	416.	343.95	{69., 588.}	40.9832
19	R0	{1.46006, 8.11496, 0.1}	{2.0776, 15.2649, 0.3}	{{1.45613, 10.8657, 0.}, {2.66225, 19.3966, 1.}}	{44.8016, 89.7967, 354.}	inf	12.	8.80432	{2.56188, 16.3994}	45.3224	0.292329	newly	15.	5.24	{1., 10.}	65.3333	0.589035	Reff	1.43232	2.04349	{1.44428, 2.59996}	45.0644	ar	496.	720.56	{108., 914.}	65.2218
20	R0	{1.14273, 6.71261, 0.1}	{1.30949, 6.29626, 0.84}	{{1.06738, 2.19254, 0.}, {1.56048, 16.8028, 3.}}	{17.6509, 68.5897, 872.}	inf	10.	7.85893	{1.97822, 14.3387}	47.8828	0.291917	newly	11.	14.51	{3., 31.}	80.6364	0.320561	Reff	1.10273	1.26603	{1.03209, 1.51335}	17.7058	ar	362.	311.58	{32., 632.}	57.4696
21	R0	{1.42432, 8.23905, 1.}	{1.16535, 2.72344, 0.08}	{{1.06738, 2.19254, 0.}, {1.25474, 3.26445, 0.}}	{18.1818, 66.9447, 92.}	inf	12.	12.041	{3.22998, 22.6987}	48.988	0.245178	newly	16.	30.92	{7., 61.}	115.25	0.331146	Reff	1.21067	0.990413	{0.904692, 1.06964}	18.1931	ar	405.	255.35	{148., 400.}	38.9951
22	R0	{1.29386, 17.863, 2.}	{1.83344, 13.9472, 0.41}	{{1.33937, 8.13911, 0.}, {2.24732, 19.0665, 1.}}	{43.1269, 24.6685, 80.5}	inf	15.	8.1477	{2.99446, 14.1305}	50.4496	0.359248	newly	3.	5.26	{1., 9.}	104.667	0.310688	Reff	1.26151	1.79937	{1.3183, 2.20967}	43.93	ar	488.	644.53	{86., 873.}	55.9611
23	R0	{1.39274, 13.0167, 5.}	{1.55047, 11.575, 1.95}	{{1.06738, 2.19254, 0.}, {1.87294, 19.0269, 3.}}	{19.7256, 40.7117, 61.}	inf	20.	40.3862	{7.04597, 67.6856}	126.09	0.381675	newly	9.	27.86	{12., 45.}	216.	0.468731	Reff	1.22283	1.32443	{0.956942, 1.57037}	16.3651	ar	475.	544.36	{160., 759.}	45.4526
24	R0	{1.32178, 15.8991, 1.}	{1.45755, 11.9994, 0.95}	{{1.10202, 5.30671, 0.}, {1.79436, 19.1609, 2.}}	{21.6622, 34.2898, 83.}	inf	13.	12.7533	{3.97727, 22.0614}	46.1254	0.247234	newly	5.	8.72	{3., 16.}	92.4	0.272168	Reff	1.27023	1.39942	{1.06417, 1.72544}	21.1398	ar	471.	464.1	{80., 748.}	47.9533
25	R0	{1.37568, 17.8427, 1.}	{1.61248, 14.5079, 1.32}	{{1.43451, 13.3174, 1.}, {1.84468, 19.9256, 2.}}	{17.2134, 23.041, 44.}	inf	15.	37.883	{25.349, 59.2546}	153.443	0.382863	newly	9.	20.14	{12., 28.}	124.444	0.330409	Reff	1.2615	1.44059	{1.29326, 1.6074}	14.2948	ar	398.	629.11	{502., 753.}	58.6759
26	R0	{1.20993, 6.4656, 0.1}	{1.76359, 13.7305, 1.26}	{{1.42801, 9.02643, 0.}, {2.036, 18.765, 2.}}	{45.7596, 113.342, 1194.}	inf	16.	33.2848	{15.4383, 56.757}	115.173	0.306992	newly	14.	20.23	{9., 32.}	61.7857	0.218747	Reff	1.13007	1.61383	{1.33359, 1.84737}	42.8072	ar	345.	697.98	{555., 828.}	103.554
27	R0	{1.20548, 13.8003, 0.1}	{1.61841, 16.3108, 0.79}	{{1.21457, 10.1856, 0.}, {1.76949, 19.2972, 2.}}	{34.9424, 27.6318, 762.}	inf	23.	12.8755	{4.52757, 19.5491}	44.5614	0.313577	newly	9.	6.01	{2., 10.}	40.1111	0.282426	Reff	1.15003	1.55978	{1.1784, 1.70361}	36.1513	ar	350.	604.48	{319., 742.}	86.5143
28	R0	{1.3176, 14.6594, 1.}	{1.4788, 13.2252, 0.59}	{{1.08887, 8.14942, 0.}, {2.2194, 19.1478, 2.}}	{25.0934, 25.3347, 75.}	inf	4.	4.34704	{1.37411, 8.45618}	62.3185	0.258472	newly	1.	2.65	{0., 6.}	193.	0.466712	Reff	1.30442	1.46232	{1.07918, 2.19134}	24.8706	ar	362.	351.98	{9., 827.}	85.9392
29	R0	{1.28202, 6.23047, 1.}	{1.37597, 8.02695, 1.01}	{{1.06738, 2.19254, 0.}, {1.85782, 17.7016, 3.}}	{16.4125, 91.3406, 105.}	inf	7.	13.2848	{1.95723, 30.1206}	131.539	0.385823	newly	13.	15.	{3., 26.}	62.1538	0.294004	Reff	1.22561	1.30397	{1.02183, 1.74352}	15.4336	ar	391.	345.61	{41., 762.}	63.5166
30	R0	{1.28177, 8.83252, 1.}	{1.59502, 10.1515, 0.99}	{{1.10202, 5.30671, 0.}, {2.26107, 17.2462, 2.}}	{33.4065, 45.0549, 83.}	inf	15.	11.3998	{2.48492, 23.7427}	49.9576	0.316244	newly	15.	9.23	{3., 17.}	47.5333	0.329964	Reff	1.24332	1.5502	{1.0755, 2.20171}	33.0992	ar	427.	483.14	{26., 849.}	72.2061
31	R0	{1.35886, 5.97708, 0.1}	{2.07073, 15.3371, 1.74}	{{1.7434, 11.3493, 1.}, {2.45321, 19.391, 2.}}	{52.3872, 156.615, 1648.}	inf	20.	42.0467	{21.7298, 69.667}	113.177	0.298118	newly	20.	24.11	{13., 37.}	41.65	0.154372	Reff	1.27733	1.89736	{1.60735, 2.18213}	48.5417	ar	491.	796.85	{695., 887.}	62.2912
32	R0	{1.27601, 17.9804, 3.}	{1.71508, 17.2536, 0.37}	{{1.4283, 12.1984, 0.}, {1.98971, 19.842, 1.}}	{34.5918, 15.3914, 87.6667}	inf	14.	10.1739	{3.9357, 16.1903}	38.8558	0.244013	newly	3.	4.93	{1., 10.}	99.	0.347617	Reff	1.24028	1.67322	{1.40001, 1.94357}	35.0876	ar	445.	618.74	{65., 813.}	58.2697
33	R0	{1.3368, 11.3235, 0.1}	{1.5834, 12.1149, 1.11}	{{1.13726, 5.71232, 0.}, {1.99753, 18.7084, 2.}}	{23.863, 40.3085, 1082.}	inf	17.	15.6798	{5.51657, 25.7518}	41.8454	0.224807	newly	9.	10.92	{5., 20.}	51.3333	0.192386	Reff	1.27531	1.51071	{1.09683, 1.88697}	23.5318	ar	417.	548.55	{91., 809.}	59.6475
34	R0	{1.21021, 11.3635, 2.}	{1.17686, 4.21503, 0.34}	{{1.03281, 2.5149, 0.}, {1.28596, 5.30671, 2.}}	{8.47384, 63.88, 84.}	inf	11.	8.97028	{2.6311, 15.5512}	44.3892	0.258201	newly	11.	17.71	{5., 30.}	89.9091	0.281471	Reff	1.12671	1.09779	{0.96635, 1.20396}	8.44688	ar	327.	220.73	{66., 444.}	52.3333
35	R0	{1.53382, 10.8866, 0.1}	{1.9104, 14.9486, 0.33}	{{1.38427, 8.26611, 0.}, {2.37462, 19.4475, 1.}}	{29.523, 47.0653, 376.}	inf	11.	13.6023	{6.04708, 23.6683}	49.0744	0.193757	newly	11.	8.01	{3., 15.}	43.5455	0.292869	Reff	1.49547	1.85631	{1.34618, 2.3154}	28.9954	ar	476.	725.18	{436., 881.}	62.4202
36	R0	{1.20423, 6.83844, 0.1}	{1.7318, 8.35387, 0.49}	{{1.06738, 2.19254, 0.}, {2.48947, 18.765, 1.}}	{47.6736, 79.4962, 518.}	inf	6.	15.0468	{4.09817, 26.9132}	163.914	0.37501	newly	7.	23.16	{5., 47.}	240.	0.434657	Reff	1.18255	1.68126	{1.0479, 2.41115}	46.1101	ar	416.	565.78	{45., 892.}	74.0481
37	R0	{1.43629, 10.1548, 3.}	{1.19368, 4.19479, 0.7}	{{1.06256, 2.19254, 0.}, {1.31748, 5.71232, 2.}}	{19.2986, 64.3848, 78.}	inf	10.	12.0649	{3.22639, 22.4171}	62.1369	0.26226	newly	5.	24.29	{5., 53.}	393.	0.583985	Reff	1.34006	1.11069	{0.985249, 1.22499}	19.2744	ar	367.	252.4	{70., 475.}	48.2997
38	R0	{1.37504, 8.93428, 1.}	{1.67565, 9.18254, 0.9}	{{1.10202, 3.02112, 0.}, {2.45321, 19.255, 2.}}	{31.6112, 58.4913, 86.}	inf	15.	14.8438	{4.35586, 27.2986}	49.7756	0.255183	newly	14.	18.48	{6., 39.}	75.2857	0.290784	Reff	1.32829	1.61529	{1.0715, 2.3621}	30.9687	ar	483.	539.33	{59., 891.}	55.5797
39	R0	{1.51637, 9.36742, 1.}	{1.20671, 3.2592, 0.18}	{{1.06738, 2.19254, 0.}, {1.28596, 3.98513, 0.}}	{21.4439, 68.6871, 102.}	inf	28.	16.158	{2.28083, 30.1239}	52.7611	0.42757	newly	20.	37.33	{6., 71.}	119.95	0.386868	Reff	1.37686	1.10906	{0.978384, 1.19903}	20.2239	ar	441.	272.02	{72., 488.}	44.6213
40	R0	{1.23658, 9.82869, 1.}	{2.52746, 10.4033, 0.41}	{{1.74592, 5.83416, 0.}, {2.9884, 13.5542, 1.}}	{104.409, 24.9551, 73.}	inf	23.	27.1191	{9.61296, 41.3782}	46.613	0.207545	newly	14.	31.99	{11., 55.}	138.357	0.342656	Reff	1.18712	2.41355	{1.69352, 2.83248}	103.312	ar	414.	868.65	{726., 943.}	110.635
41	R0	{1.24247, 5.4242, 0.1}	{1.91782, 9.92334, 1.16}	{{1.21159, 2.5364, 0.}, {2.8186, 19.255, 3.}}	{57.0511, 114.119, 1146.}	inf	9.	12.4198	{4.65405, 21.329}	62.0328	0.217938	newly	11.	21.68	{3., 45.}	136.545	0.372688	Reff	1.22135	1.87678	{1.18553, 2.74091}	56.4053	ar	345.	615.96	{66., 921.}	105.751
42	R0	{1.28208, 4.40223, 0.1}	{2.33291, 11.7137, 0.8}	{{1.65456, 4.06661, 0.}, {2.91001, 17.1903, 2.}}	{82.6754, 170.14, 794.}	inf	3.	21.3096	{6.81042, 34.6922}	611.866	0.787083	newly	6.	21.11	{6., 43.}	256.167	0.482577	Reff	1.26028	2.24756	{1.60602, 2.79794}	79.1171	ar	339.	825.76	{608., 937.}	146.289
43	R0	{1.56773, 6.50474, 0.1}	{2.06952, 7.80931, 0.61}	{{1.24751, 2.56072, 0.}, {2.80569, 15.146, 2.}}	{40.1221, 56.8311, 636.}	inf	6.	7.23287	{2.39857, 14.0007}	59.9289	0.239006	newly	5.	21.25	{2., 31.}	347.8	0.480301	Reff	1.55205	2.04501	{1.24045, 2.7743}	39.815	ar	498.	701.83	{212., 926.}	64.4438
44	R0	{1.10489, 6.01941, 1.}	{1.32548, 9.67408, 1.53}	{{1.10202, 3.26445, 0.}, {1.6004, 16.157, 3.}}	{20.2729, 76.9521, 145.}	inf	18.	14.9457	{4.79675, 26.9011}	45.6737	0.268031	newly	22.	12.92	{5., 19.}	54.8182	0.357572	Reff	1.03418	1.24307	{1.0467, 1.48978}	20.3923	ar	346.	359.85	{62., 635.}	58.3555
45	R0	{1.25888, 14.1926, 1.}	{1.17835, 6.47897, 0.47}	{{1.10202, 5.30671, 0.}, {1.41689, 12.6439, 2.}}	{12.6046, 57.0571, 101.}	inf	42.	15.8155	{3.33135, 31.6145}	67.4186	0.587179	newly	17.	16.36	{3., 29.}	47.4118	0.262528	Reff	1.09523	1.05393	{0.984903, 1.2261}	10.0442	ar	471.	245.68	{99., 425.}	54.0807
46	R0	{1.45292, 16.616, 2.}	{1.56528, 14.2938, 0.52}	{{1.12882, 8.84894, 0.}, {2.2194, 19.5242, 1.}}	{21.883, 22.5919, 76.}	inf	9.	4.02478	{1.33525, 8.4739}	61.0656	0.470231	newly	4.	2.42	{0., 5.}	62.5	0.556747	Reff	1.43258	1.55035	{1.11778, 2.18349}	21.8402	ar	494.	423.71	{7., 813.}	62.3988
47	R0	{1.22528, 16.6033, 0.1}	{1.49893, 12.5584, 0.47}	{{1.07342, 7.26549, 0.}, {2.00049, 18.727, 1.}}	{28.7115, 28.7211, 492.}	inf	12.	5.68561	{1.54806, 10.4465}	60.8295	0.469235	newly	3.	3.74	{1., 8.}	76.	0.35574	Reff	1.19955	1.47572	{1.06255, 1.96919}	28.7767	ar	368.	392.06	{13., 817.}	83.9511
48	R0	{1.1669, 6.92715, 0.1}	{1.29019, 6.02421, 0.91}	{{1.06738, 2.19254, 0.}, {1.68078, 15.6632, 3.}}	{15.8555, 73.1604, 944.}	inf	9.	19.0002	{4.10949, 37.2163}	133.156	0.35027	newly	10.	29.91	{8., 58.}	210.1	0.424948	Reff	1.05138	1.14754	{0.955931, 1.46464}	14.5319	ar	416.	344.65	{101., 661.}	47.4111
49	R0	{1.32749, 16.0887, 3.}	{1.648, 13.4118, 0.5}	{{1.27357, 8.26611, 0.}, {2.11476, 19.3966, 1.}}	{26.8487, 24.6351, 84.}	inf	11.	10.5444	{2.80607, 20.6198}	48.2496	0.253557	newly	7.	6.06	{2., 11.}	44.5714	0.287211	Reff	1.29165	1.60302	{1.2498, 2.01938}	26.6729	ar	353.	559.45	{34., 815.}	87.1246
50	R0	{1.29636, 15.2609, 0.1}	{2.06556, 17.0524, 0.16}	{{1.79436, 13.7237, 0.}, {2.27582, 19.0665, 1.}}	{59.3352, 17.9883, 232.}	inf	39.	57.9535	{35.7571, 73.8006}	52.3538	0.173636	newly	16.	27.67	{17., 37.}	73.6875	0.224311	Reff	1.16283	1.81123	{1.6001, 1.98832}	55.76	ar	470.	804.67	{711., 874.}	71.2064
